# Management of chronic hepatitis B patients: Efficacy & limitation of nucleos(t)ide analogues

**Published:** 2011-01

**Authors:** Jun Inoue, Yoshiyuki Ueno, Tooru Shimosegawa

**Affiliations:** Division of Gastroenterology, Tohoku University Graduate School of Medicine, Sendai, Japan

Hepatitis B virus (HBV) causes a spectrum of liver diseases including acute hepatitis, chronic hepatitis, liver cirrhosis, and hepatocellular carcinoma. HBV contains a circular, partially double-stranded DNA genome of 3.2 kb. This genome includes 4 partly overlapping open reading frames. One of these is the polymerase gene that encodes for the polymerase protein including the reverse transcriptase (RT) region. In the process of HBV replication, the pregenomic RNA, which is transcribed from covalently closed circular DNA (cccDNA), is reverse-transcribed by the polymerase protein of HBV. This step is mainly targeted by nucleos(t)ide analogues such as lamivudine, adefovir, entecavir, tenofovir, and telbivudine. Oral administration of these drugs results in virological, biochemical, and histological improvement in most patients^1^, but the effect is often transient due to the emergence of drug-resistant mutants of HBV.

In this issue, Kumar *et al*^2^ performed a randomized pilot study to compare lamivudine and adefovir in terms of the HBV kinetics. To our knowledge, this is the first report of a randomized study comparing these drugs, and hence valuable. Based on several previous studies, it was thought that adefovir has weaker suppressive effect on serum HBV DNA than other nucleos(t)ide analogues including lamivudine^3^,^4^: the virological response (undetectable HBV DNA) rate of adefovir at 1 year was 21 per cent, and that of lamivudine was 39-44 per cent. However, this 24-wk study showed no significant difference in virological, biological, and histological responses between adefovir and lamivudine. A recent meta-analysis, which compared 48-52-wk outcomes of several antiviral drugs to HBV, demonstrated that there was no significant difference in outcomes between adefovir and lamivudine in both HBeAg-positive and HBeAg-negative patients^5^. Therefore, the results of Kumar *et al*^2^ seem to be reasonable. The sample size however is small and the treatment duration was short, as they described. A larger samples is needed to reach a conclusion, and analyses should be performed in view of the HBeAg status and HBV genotypes, which are known to affect the efficacy of anti-viral drugs^6^.

The monotherapy of lamivudine or adefovir is associated with the highly frequent emergence of drug-resistant mutations after long treatment (70 and 29% at year 5, respectively^3^), and present American Association for the s0 tudy of Liver d0 iseases (AASLD)^4^ and European Association for the s0 tudy of the Liver (EASL)^3^guidelines do not recommend these drugs for treatment-naïve patients. For these patients, entecavir or tenofovir are potent and rarely result in drug-resistant mutants (1.2% at year 5 and 0% at year 1, respectively^3^), and long-term continuation of the drugs without drug resistance has become possible for most patients. As long-term administration of these drugs can be an economic burden for a substantial number of patients, both the cost and efficacy should be considered in the choice of drugs. The replacement of methionine at amino acid 204 to valine or isoleucine (rtM204V/I) within the tyrosine-methionine-aspartate-aspartate (YMDD) motif in the reverse transcriptase region of HBV polymerase is found in most lamivudine-resistant patients. The management of these patients is still a major problem. Previous *in vitro* studies demonstrated that adefovir and entecavir have a suppressive effect on lamivudine-resistant mutants^7^. A combination therapy of lamivudine and adefovir is found superior to adefovir monotherapy for lamivudine-resistant patients^8^. Because a pre-existing YMDD mutation predisposes the emergence of entecavir resistance^7^, entecavir monotherapy is less attractive for lamivudine-resistant patients.

Because HBV polymerase lacks a conventional proofreading function, the mutation rate of HBV is much higher than other DNA viruses. Naturally-occurring HBV mutations are reported to affect the disease outcome, including cirrhosis, hepatocellular carcinoma, and fulminant hepatitis^9^. Drug-resistant mutations can occur in treatment-naïve patients and, interestingly, recent reports from Japan^10^ and China^11^ described that drug-resistant mutants were found in acute hepatitis B patients (4 and 7%, respectively). Although the drug-resistant mutants are in general thought to have less replication capacity than the wild type, the mutants are transmissible.

Although nucleos(t)ide analogues effectively decrease HBV DNA in serum, cccDNA may not be eliminated in the liver. If the treatment is discontinued halfway, it can be a template for HBV replication. Intrahepatic HBV DNA persists even in patients who have lost serum HBsAg^12^. The amount of cccDNA can be decreased by antiviral drugs, and the degree of reduction was reported to predict the sustained response to therapy^13^. Kumar *et al*^2^ quantified intrahepatic HBV DNA at the start and end of 6-months therapy, but not cccDNA. The intrahepatic HBV DNA includes cccDNA and other forms of HBV DNA such as single-stranded DNA, double-stranded linear DNA, and relaxed circular DNA. Because it was reported that there was a correlation between the level of intrahepatic HBV DNA and cccDNA^13^, the reduction of intrahepatic HBV DNA in this study might also demonstrate a reduction of cccDNA. The investigation of agents that eliminate cccDNA in the liver is a great challenge in HBV research.

One of the problems in the treatment of chronic HBV infection is the lack of clearly defined end points. The loss of HBsAg is thought to be the best end point clinically, but it rarely occurs. The level of hepatitis B core-related antigen (HBcrAg) in the serum, which is associated with the level of intrahepatic cccDNA, was described as predicting a sustained response^14^. The seroconversion of HBeAg has been thought to be one of the major signs of effective suppression of HBV for HBeAg-positive patients. However, Reijnders *et al* 15 reported recently that the seroconversion induced by nucleos(t)ide analogues was transient in most cases. They described that the long-term continuation of these drugs, irrespective of the occurence of HBeAg seroconversion, appears to be necessary. The end points of the therapies have to be discussed further.

The development of antiviral agents for HBV has progressed successfully, and currently there are many therapeutic options. Interferon-α and pegylated interferon-α can be effective, although the details are not described here. However, at present, there is no therapy that can eradicate HBV completely. There is increasing evidence that profound, durable therapeutic suppression of HBV DNA results in slowing and reversing the progression of chronic HBV infection. Once the drug-resistant mutations occur, the management of patients become difficult. Therefore, more potent and less resistance-prone antiviral drugs are needed for the initial therapy. There is a possibility that more effective drugs or combination therapies, which can reduce intrahepatic cccDNA effectively, will be able to shorten the therapy period in the future.
